# The Network Mechanism of the Central Circadian Pacemaker of the SCN: Do AVP Neurons Play a More Critical Role Than Expected?

**DOI:** 10.3389/fnins.2019.00139

**Published:** 2019-02-25

**Authors:** Michihiro Mieda

**Affiliations:** Department of Integrative Neurophysiology, Graduate School of Medical Sciences, Kanazawa University, Kanazawa, Japan

**Keywords:** circadian rhythm, suprachiasmatic nucleus, vasopressin, vasoactive intestinal peptide, neural network

## Abstract

The suprachiasmatic nucleus (SCN) functions as the central circadian pacemaker in mammals and entrains to the environmental light/dark cycle. It is composed of multiple types of GABAergic neurons, and interneuronal communications among these neurons are essential for the circadian pacemaking of the SCN. However, the mechanisms underlying the SCN neuronal network remain unknown. This review will provide a brief overview of the current knowledge concerning the differential roles of multiple neuropeptides and neuropeptide-expressing neurons in the SCN, especially focusing on the emerging roles of arginine vasopressin-producing neurons uncovered by recent studies utilizing neuron type-specific genetic manipulations in mice.

## Introduction

The circadian oscillator of the hypothalamic suprachiasmatic nucleus (SCN) is the central pacemaker in mammals, orchestrating multiple circadian biological rhythms in the organism and being regulated according to the external light/dark conditions conveyed from the eye (Reppert and Weaver, 2002). The SCN contains ∼20,000 neurons, most of which are able to oscillate autonomously. Individual cellular oscillators (cellular clocks) are driven by autoregulatory transcriptional/translational feedback loops (TTFLs) of clock genes in concert with cytosolic signaling molecules, including cAMP and Ca^2+^ (Welsh et al., 2010; Herzog et al., 2017; Takahashi, 2017). Surprisingly, these intracellular molecular mechanisms are not unique to SCN cells but are shared with peripheral cells (Balsalobre et al., 1998). Rather, intercellular communications among SCN cells through the neuronal and diffusible network are the unique feature of the SCN that is responsible for the generation of highly robust and coherent oscillations as an ensemble (Welsh et al., 2010).

## Structure of the SCN

**FIGURE 1:**
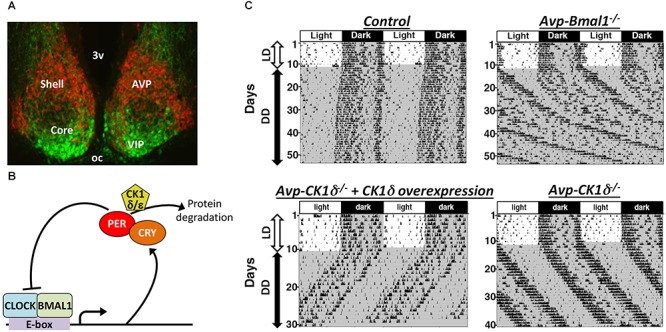
The SCN is composed of multiple types of clock neurons. **(A)** A coronal section of mouse SCN, showing the dorsal shell region delineated by the expression of tdTomato fluorescent protein in AVP neurons (red) and the ventral core region containing VIP neurons labeled immunofluorescently (green). 3v, third ventricle; oc, optic chiasm. **(B)** A simplified schematic of cellular clock, the molecular machinery of circadian clock composed of TTFLs of clock genes. Only the part relevant to this review is shown here. For the detailed mechanism, please see other reviews, such as Takahashi, 2017. Transcription factors CLOCK and BMAL1 bind to E-box sequences to drive expression of PER and CRY, which in turn suppress CLOCK-BMAL1 activity, closing a negative feedback loop. Phosphorylation of PER proteins by *CK1δ* enhances those proteins’ degradation and consequently accelerates the speed of cellular clocks. **(C)** Representative actograms of mice with AVP neuron-specific genetic manipulations. The free-running period is lengthened in both *Avp-Bmal1^*-/-*^* and *Avp-CK1δ^*-/-*^* mice, while the activity time was lengthened only in *Avp-Bmal1^*-/-*^* mice. Modified from Mieda et al. (2015, 2016).

The SCN is a heterogeneous structure that consists of multiple types of GABAergic neurons (Antle and Silver, 2005). Many of them co-express neuropeptides, represented by. arginine vasopressin (AVP)-producing neurons located in the shell, the dorsomedial part, of the SCN, as well as by vasoactive intestinal peptide (VIP)-producing neurons and gastrin releasing peptide (GRP)-producing neurons in the core, the ventrolateral part, of the SCN (Figure 1A). Rhythmic *Period* (*Per*) expression in constant darkness (DD) is highest in the SCN shell (Hamada et al., 2004). In contrast, the SCN core contains retinorecipient neurons that respond immediately to the environmental light stimuli and communicate this information to the shell (Silver et al., 1996; Shigeyoshi et al., 1997). Two other input pathways from the median raphe and intergeniculate leaflet also terminate mainly in the SCN core, while afferents from the hypothalamus and limbic areas terminate mainly in the SCN shell (Moga and Moore, 1997). SCN neurons project principally to areas within the diencephalon, especially to the subparaventricular zone, the area just dorsal to the SCN. The majority of efferent projections originates in the shell, but the core neurons also send efferent projections in a manner different from the shell neurons (Leak and Moore, 2001). Within the SCN, core neurons send projections densely to the shell, while fibers of shell neurons in the core are sparse (Leak et al., 1999).

## Vip: a Critical Synchronizer of SCN Neurons

Vasoactive intestinal peptide has been demonstrated to be especially important for the maintenance and synchronization of cellular clocks in the individual SCN neurons (Herzog et al., 2017). Thus, mice lacking *Vip* or VIP receptor *Vipr2* gene demonstrate drastically weakened behavioral rhythms, often with multiple period components (Harmar et al., 2002; Colwell et al., 2003; Aton et al., 2005). At the cellular level, circadian oscillations of electrical firing and clock gene expression of individual SCN neurons (cellular clocks) are desynchronized in slices (Aton et al., 2005; Maywood et al., 2006; Brown et al., 2007). Furthermore, the numbers of rhythmic neurons are drastically reduced (Aton et al., 2005; Maywood et al., 2006; Brown et al., 2007). These observations suggest that VIP functions as a master synchronizer in the SCN.

Consistent with these observations, optogenetic activation of VIP neurons in the SCN phase-shifts the cellular clock (circadian PER2::LUC oscillation) in explants and entrains the behavior rhythm *in vivo* (Jones et al., 2015; Mazuski et al., 2018). In addition, chemogenetic inhibition of these neurons attenuates the light-induced phase-shift of circadian behavior rhythm (Jones et al., 2018). In explants, prolonged chemogenetic stimulation of these neurons further reprograms the global spatiotemporal dynamics of the SCN cellular clocks (Brancaccio et al., 2013).

On the other hand, VIP neurons may not play a significant role in the pacemaking of circadian rhythms by the SCN network, that is, determination of the circadian period, as discussed again later. Lee et al. demonstrated that overexpression of the *Clock^Δ*19*^* transgene in VIP neurons had no effect on the behavioral free-running period, although such a *Clock^Δ*19*^* overexpression lengthens the intrinsic period of cellular clocks in the manipulated cells (Table 1; Lee et al., 2015). These results contrasted clearly with the observations that the same genetic manipulations in SCN neurons expressing a neuropeptide neuromedin-S (NMS) lengthens the period of behavior rhythm: NMS neurons include VIP neurons, AVP neurons, and some other types of neurons. In conjunction with the observation that genetic ablation of cellar clocks specifically in NMS neurons disrupts circadian rhythms, Lee et al. (2015) concluded that NMS neurons act as essential pacemakers in the SCN.

**Table 1:** Effects of neuron type-specific alterations of cellular circadian periods on the free-running periods of circadian behavior rhythms.

Manipulated cells	Composition of manipulated cells	Change in behavior period	Behavior period in controls	Genetic manipulation	Control mice	Crossed mice/ Injected AAV	Name in original papers	References
NMS neurons	AVP neurons, VIP neurons, other neurons	∼60 min longer	∼24 h	*Clock*^Δ*19*^ ovcroxpression	*R26-Clock^Δ*19*^*^∗^	*Nms-iCre* mice	*Nms-Clock^Δ*19*^*	Lee et al., 2015
VIP neurons	VIP neurons	no change	∼24 h	*Clock^Δ*19*^* overexpression	*R26-Clock^Δ*19*^*^∗^	*Vip-ires-Cre* mice	*Vip-Clock^Δ*19*^*	Lee et al., 2015
AVP neurons	AVP neurons	∼50 min longer	∼24 h	*CKI δ* deletion	*CK1 δ^flox/flox^*	*Avp-iCre* mice	*Avp-CK1 δ^*-/-*^*	Mieda et al., 2016
GABA neurons	Most SCN neurons	∼40 min longer	∼24 h	*CKI δ* deletion	*CKI δ^flox/flox^*	*Vgat-ires-Cre* mice	*Vgat-Cre^+^ CK1 δ^fl/fl^*	van der Vinne et al., 2018
Drdla neurons	62% of AVP neurons, 81% of VIP neurons, other neurons	∼4 h longer in 60%, no change in 33%	∼20 h	*CKI 𝜖^Tau^* deletion	*CKI 𝜖^flox-Tau/flox-Tau#^*	*Drd 1a-Cre* mice	*DCR^+^/CK1𝜖^Tau/Tau^*	Smyllie et al., 2016
SCN neurons	SCN neurons	∼4 h longer	∼20 h	*CKI 𝜖^Tau^* deletion	*CKI 𝜖^flox-Tau/flox-Tau^*	*Syn-mCherry::Cre* AAV	*CK1𝜖^Tau/Tau^+Syn-m Cherry::Cre*	Brancaccio et al., 2017
SCN astroeytes	SCN astrocytes	∼4 h longer	∼20 h	*CKl 𝜖^Tau^* deletion	*CKI 𝜖^flox-Tau/flox-Tau^*	*GFAP-m Cherry::Cre* AAV	*CK1𝜖^Tau/Tau^+GFAP-m Cherry::Cre*	Brancaccio et al., 2017


*^∗^Rosa26-fioxed stop-tTA;TRE-Clock^Δ19^ mice. ^#^also contains a Rosa26-floxed stop-EYFP allele.*

## AVP Peptide May Also Be Involved in the Coupling of the SCN Neurons

The concentration of AVP in the cerebrospinal fluid (CSF) daily fluctuates with a peak in the morning (Stark and Daniel, 1989; Kalsbeek et al., 2010). Such a circadian variation of CSF AVP level has been shown to originate from the AVP content in the SCN (Södersten et al., 1985). Indeed, the transcription of *Avp* gene in the SCN is under the control of cellular clocks, the molecular machinery of the circadian clock composed of TTFL of clock genes (Jin et al., 1999). In conjunction with the fact that AVP-deficient Brattleboro rats display attenuated circadian rhythms but little abnormality in circadian pacemaking, AVP has been considered to function as an SCN output (Groblewski et al., 1981; Brown and Nunez, 1989; Kalsbeek et al., 2010). A recent optogenetic study directly demonstrated clock-driven AVP neurotransmission that mediates anticipatory thirst prior to sleep (Gizowski et al., 2016).

Nevertheless, AVP may also play a minor but significant role in the coupling of SCN neurons. In coculture experiments of SCN explants, the requirement of AVP signaling for the synchronization of SCN neurons becomes manifest in the absence of VIP signaling (Maywood et al., 2011; Edwards et al., 2016; Ono et al., 2016). Deletion of V1a receptor, the principal AVP receptor of the SCN, lengthens the activity time in DD by approximately 100 min in mice, suggesting the attenuated coupling among SCN neurons (Li et al., 2009). A small number of these mice even show arrhythmicity. In another study, *V1a^*-/-*^*; *V1b^*-/-*^* mice were reported to immediately reentrain to phase-shifted LD cycles whereas their free-running rhythms are intact, indicating that interneuronal communication mediated by AVP make the SCN resistant to environmental perturbations such as jet lag (Yamaguchi et al., 2013). In contrast to VIP, *Avp* knockout mice are not available for the examination of circadian behavior rhythm, because they do not survive beyond postnatal day 7 (Yoshikawa et al., 2015). Therefore, development and study of SCN-specific knockout mice would further elucidate the physiological role of AVP signaling in the central circadian clock.

## AVP-Producing “Neurons” Are Critical for the Coupling of SCN Neurons

Arginine vasopressin neurons express neurotransmitters other than AVP, such as GABA and prokineticin 2 (Antle and Silver, 2005; Masumoto et al., 2006; Welsh et al., 2010). Multiple transmitters in one neuronal type may transmit differential information, as reported in orexigenic AgRP neurons in the hypothalamic arcuate nucleus and wake-stabilizing orexin neurons in the lateral and perifornical hypothalamus (Krashes et al., 2013; Muschamp et al., 2014; Schöne et al., 2014). Therefore, neurons producing AVP may play a more fundamental role in the circadian pacemaking of the SCN than the AVP molecule does. This hypothesis was testable by genetically manipulating AVP neurons using the Cre-loxP system. When *Bmal1*, an essential transcription factor of cellular clocks (Figure 1B; Bunger et al., 2000), was deleted specifically in AVP neurons (*Avp-Bmal1^*-/-*^* mice), mice demonstrated a significant impairment of the locomotor activity rhythm in DD (Figure 1C; Mieda et al., 2015). When released into DD, the interval between the activity onset and offset (activity time) gradually became expanded by approximately 5 h as compared with that in controls. Their free-running period was approximately 50 min longer on average than that of control mice. Furthermore, a small number of *Avp-Bmal1^*-/-*^* mice even demonstrated arrhythmicity. Importantly, *Bmal1* restoration in AVP neurons of the SCN with the aid of a recombinant AAV vector reversed the circadian impairment of *Avp-Bmal1^*-/-*^* mice almost completely. These results indicate that the cellular circadian oscillation persists, but the mutual coupling between clock neurons regulating the onset and offset components of activity may be severely impaired in the SCN of *Avp-Bmal1^*-/-*^* mice.

In these mice, the circadian expression of factors involved in intercellular communications, including *Avp*, *Prokineticin 2*, and *Rgs16*, was considerably decreased in the SCN shell, where AVP neurons are located. In SCN explants, PER2::LUC oscillations in the shell cells were attenuated with highly variable and lengthened periods. Collectively, *Bmal1*-based cellular clocks of AVP neurons are likely to enhance the coupling of the SCN cells to generate robust circadian rhythms by regulating expression of multiple factors involved in interneuronal communications (Mieda et al., 2015).

## Avp Neurons Are Involved in the Circadian Period Determination at the Network Level

By artificially manipulating the period of cellular clocks specifically in AVP neurons, the possibility that AVP neurons actively work as pacemaker cells to determine the period of circadian rhythm generated by the SCN network was examined (Mieda et al., 2016). It has been shown that the phosphorylation of PER proteins by casein kinase 1δ (CK1δ) regulates the speed of cellular clocks (Figure 1B; Herzog et al., 2017). Artificial lengthening of the cellular circadian period specifically in AVP neurons, achieved by deleting *CK1δ* in AVP neurons (*Avp-CK1δ^-/-^*), also lengthened the free-running period of behavior rhythm by approximately 50 min, while their activity time remained normal (Figure 1C and Table 1). Conversely, artificial shortening of the AVP neuronal circadian period, archived by overexpression of *CK1δ1* in these neurons via focal injection of a Cre-dependent AAV expression vector, shortened the period of behavior rhythm (Figure 1C). Thus, the manipulation of *CK1δ* expression levels in AVP neurons of the SCN bidirectionally changed the free-running period of behavior rhythm, suggesting that AVP neurons do indeed regulate SCN pacemaking (Mieda et al., 2016).

How much do AVP neurons contribute to the period determination? Mice in which *CK1δ* was deleted in the entire SCN, using GABAergic neuron-specific *Vgat-Cre* driver mice, also showed a lengthened free-running period of behavior rhythm by approximately 40 min (Table 1; van der Vinne et al., 2018), which was comparable to that in *Avp-CK1δ^*-/-*^* mice. These data indicate that AVP neurons are the principal determinant of circadian period generated by the SCN network *in vivo*.

Because of the coherently lengthened free-running period of *Avp-CK1δ^*-/-*^* mice (Figure 1C), the cellular clocks (PER2::LUC oscillations) of the entire SCN were also expected to oscillate with a longer period in slices. Contrary to such an expectation, however, the SCN shell and core of *Avp-CK1δ^*-/-*^*; *Per2::Luc* mice transiently demonstrated different cellular periods in explants (Mieda et al., 2016). The period of the shell was longer, but this lengthening did not last into the subsequent cycles. These data suggest that the core modulated the shell in the prolonged SCN culture. Indeed, the lengthening of shell’s period in *Avp-CK1δ^*-/-*^*; *Per2::Luc* mice lasted for a longer duration when slices were surgically cut between the shell and core. A similar dissociation of behavior rhythm and PER2::LUC rhythm has also been observed in *Avp-Bmal1^*-/-*^* mice (Mieda et al., 2015). Thus, the intact structure of the SCN and/or its connections with other brain areas might be important for the coupling between SCN shell and core *in vivo*.

In rodents, core neurons communicate with those in the shell, while there is less communication in the reverse direction (Leak et al., 1999). A recent study of mouse SCN connectome reported that, although the direct connection from AVP neurons to VIP neurons is extremely sparse, AVP neurons make plenty of contacts onto other types of neurons in the SCN core, such as GRP neurons, raising the possibility that AVP neurons communicate well with VIP neurons indirectly via those non-VIP core neurons (Varadarajan et al., 2018). Such asymmetric anatomical interactions between the SCN core and shell may make shell-to-core interaction more fragile in slices.

## The Roles of AVP Neurons in the SCN Network

Lee et al. (2015) demonstrated that lengthening the cellular circadian period of NMS-producing SCN neurons by overexpression of *Clock^Δ*19*^* lengthened the free-running period of behavior (Table 1). Nevertheless, NMS neurons are still a heterogeneous population that contains AVP neurons, VIP neurons, and other types of neurons (Lee et al., 2015), leaving the long-standing debate on the differential roles of the shell and core of the SCN on its pacemaking unresolved.

More recently, Smyllie et al. (2016) created chimeric mice by crossing *Drd1a-Cre* mice to floxed *CK1𝜖^Tau/Tau^* mice, whose SCN contained dopamine 1a receptor (Drd1a) cells (*CK1𝜖^*-/-*^* cells) with an intrinsic cellular period of 24 h alongside non-Drd1a cells (*CK1𝜖^Tau/Tau^* cells) with a period of 20 h (Table 1). Remarkably, 60% of these mice showed 24 h periods of behavior and SCN PER2::LUC rhythms, whereas 33% showed 20 h periods. Drd1a cells contain 63% of all SCN cells, including 62% of AVP neurons and 81% of VIP neurons (Smyllie et al., 2016). The fact that the behavioral period did not necessarily follow the cellular period of 80% of VIP neurons is consistent with the earlier finding by Lee et al. (2015) that lengthening the VIP neuronal cellular period had no effect on the behavioral free-running period. Collectively, these observations suggest that VIP neurons may not be directly involved in the pacemaking of the SCN, although VIP signaling plays a principal role in the synchronization of SCN neurons.

Taken in conjunction with data indicating that AVP neurons are involved in the SCN pacemaking (Mieda et al., 2016), as described earlier, the slight difference in the ratio of 24 h AVP neurons to 20 h AVP neurons in Smyllie et al. (2016) could exert a substantial impact on the period in which the chimeric SCN oscillates. In addition, the extent of lengthening in free-running period in mice lacking *CK1δ* in the entire SCN is comparable to that in mice with AVP neuron-specific *CK1δ* deletion (van der Vinne et al., 2018), as discussed earlier. These observations suggest that AVP neurons are the primary determinant of the period of circadian rhythm generated by the SCN network. VIP neurons may play a dominant role in the synchronization and phase regulation of SCN neurons, but their contribution in period determination may be little.

Recently, striking contributions of astrocytes of the SCN in the circadian pacemaking was reported (Barca-Mayo et al., 2017; Brancaccio et al., 2017; Tso et al., 2017). SCN astrocytes and neurons are likely to act as two arms of the central circadian pacemaker network, which shows oscillations anti-phasic to each other (Brancaccio et al., 2017). These neuronal and astrocytic oscillators are coupled via glutamate released from astrocytes, which increases presynaptic GABA release and subsequently suppresses neuronal activity of postsynaptic neurons during night. Floxed *CK1𝜖^Tau/Tau^* mice that originally had a free-running behavior period of 20 h changed the period to 24 h when *CK1𝜖^Tau^* alleles were deleted specifically in SCN astrocytes via viral Cre delivery, suggesting that SCN astrocytes can control the period of circadian behavior rhythms (Table 1). Interestingly, the same reversal of free-running period was observed when *CK1𝜖^Tau^* alleles were deleted specifically in SCN neurons of the same mice. Therefore, both SCN astrocytes and neurons are equally able to impart timekeeping information to the rest of the body (Brancaccio et al., 2017). However, these results may appear a little strange and difficult to interpret. Although astrocyte- and neuron-specific deletions of *CK1𝜖^Tau^* in floxed *CK1𝜖^Tau/Tau^* mice resulted in reversed temporal misalignments of the SCN – that is, the 20 h neuronal clock and the 24 h astrocytic clock, and vice versa – the chimeric mice always showed a free-running period of 24 h. One explanation for these observations may be that cellular clocks and the SCN network are optimized to work at 24 h and therefore would be advantaged in the chimeras over the 20 h cells, regardless of which cell type has been targeted (Brancaccio et al., 2017). It would be very interesting to examine whether artificial lengthening (by *CK1δ* deletion or *Clock^Δ*19*^* overexpression) or shortening (by *CK1δ1* overexpression) of the astrocytic cellular period from 24 h alters the free-running period of behavior rhythm as much as neuronal manipulations do. In any case, comprehensive understanding of the network principle of the SCN central circadian clock needs further study.

## Concluding Remarks

A previous pioneering study utilized chimera mice of wild type and long-period *Clock^Δ*19/*Δ*19*^* mutant cells to address the network mechanism of the circadian period determination by the SCN (Low-Zeddies and Takahashi, 2001). In these mice, random subsets of wild type SCN cells were replaced with *Clock^Δ*19/*Δ*19*^* cells. The proportion of *Clock^Δ*19/*Δ*19*^* versus wild type cells largely determined circadian behavior in chimeric individuals. However, the intermediate periods were observed in some but not evident in all balanced chimeras. This fact indicates that the emergence of intermediate periods is dependent on not only the proportion but also the distribution of wild type and *Clock^Δ*19/*Δ*19*^* cells (Low-Zeddies and Takahashi, 2001), suggesting unequal contributions among SCN cells to the period determination. Cell type-specific manipulations of the cellular circadian period described earlier in this review further support such an idea that there exist cells that function as the dominant pacemaking elements in the SCN network, a likely candidate of which may be AVP neurons.

Thus, as the cellular clocks have molecular mechanisms to determine their period, amplitude, and phase within the individual cells, the SCN may have multicellular and network mechanisms to determine the period, amplitude, and phase of the circadian rhythm it generates, which is not a simple summation of multiple cellular clocks. In other words, there exists functional localization within the SCN. The characterization of *Avp-Bmal1^*-/-*^* mice and *Avp-CK1δ^*-/-*^* mice definitively demonstrated that cellular clocks of SCN AVP neurons play a critical role in the generation of robust circadian behavior rhythm through the regulation of the coupling of SCN neurons, as well as in the determination of the circadian period. Additional manipulations of cellular clocks and neuronal properties in various combinations of neuron types and genetic-engineering techniques would provide further information to comprehensively understand the principle of the SCN neural network as the central circadian pacemaker.

## Author Contributions

The author confirms being the sole contributor of this work and has approved it for publication.

## Conflict of Interest Statement

The author declares that the research was conducted in the absence of any commercial or financial relationships that could be construed as a potential conflict of interest.
